# Epigenetic silencing of miR-520c leads to induced S100A4 expression and its mediated colorectal cancer progression

**DOI:** 10.18632/oncotarget.15499

**Published:** 2017-02-18

**Authors:** Giridhar Mudduluru, Katharina Ilm, Steffen Fuchs, Ulrike Stein

**Affiliations:** ^1^ Experimental and Clinical Research Center, Charité University Medicine Berlin and Max Delbrück Center for Molecular Medicine in the Helmholtz Association, Berlin, Germany; ^2^ German Cancer Consortium (DKTK), Heidelberg, Germany

**Keywords:** colorectal cancer, metastasis, epigenetic regulation, S100A4, microRNA

## Abstract

The S100 calcium-binding protein A4 (S100A4) induces epithelial mesenchymal transition, migration, invasion, angiogenesis and metastasis. Its induced expression in several cancer types correlates with poor prognosis. Apart from the functional and transcriptional regulatory aspects of S100A4, its post-transcriptional regulation is not yet clearly elucidated. In this study, we show that microRNAs (miR) miR-505-5p and miR-520c-3p target the 3′-UTR of S100A4 and inhibits its expression and its mediated migration and invasion. 5-Aza treatment significantly increased miR-520c-3p expression and reduced the S100A4 protein amounts. The upstream promoter region of miR-520c is hypermethylated irrespective of the metastasis status of colorectal cancer (CRC) patient tissues and in all analyzed CRC cell lines. Moreover, in a cohort of CRC patient specimen (*n* = 59), miR-520c-3p was significantly downregulated. miR-520c-3p stably expressing HCT116 cells showed a reduced metastasis formation in livers after implanting in mice spleen. Taken together, our findings demonstrate that S100A4 is post-transcriptionally regulated by tumor suppressor miRs, miR-505c-5p and miR-520c-3p, and particularly miR-520c-3p expression is epigenetically silenced in CRC.

## INTRODUCTION

Among the different cancer entities, colorectal cancer (CRC) is one of the most common cancers in both men and women worldwide. According to estimations, cancer cases worldwide might raise to 24 million by 2035 from 14 million as projected in 2012. This huge increase in new cases is a challenge for both governments and health organisations, which can be better handled with early detection and better treatment methods [1, 2]. Early detection of disease is a tough and laborious process due to the heterogeneity in the causes of cancer, both genetically and environmentally. One approach to address this is the search for novel tumor markers and the study of their regulation and molecular function [3, 4]. One such molecule is S100 calcium-binding protein A4 (S100A4), which has been identified as a prognostic biomarker in a variety of solid cancers [5, 6].

S100A4 is a calcium binding protein with the mass of 10–12 kDa and belongs to the family of S100 proteins, which consists of 21 members [7, 8]. S100A4 expression is transcriptionally regulated by β-catenin and both were found to be highly expressed at the invasive margin of a tumor [9–11]. β-catenin is a downstream effector molecule of the Wnt signaling axis. This pathway is deregulated in different cancers, including CRC, due to the loss of function of the β-catenin destruction complex or gain of function of β-catenin itself [12, 13]. S100A4 is known for its ability to activate several signaling cascades, like the receptor for advanced glycation end products (RAGE) signaling, mitogen-activated protein (MAP) kinases, and activated nuclear factor kappa-light-chain-enhancer of activated B cells (NFκB) [14]. Moreover S100A4 increases the secretion of matrix metalloproteinases (MMP) such as MMP9 and MMP13 [15, 16]. Epithelial-mesenchymal transition (EMT) is one of the key switch events in cancer progression. S100A4 plays an important role in inducing proliferation, EMT, migration, invasion and colony formation [6, 17]. S100A4 binds to cytoskeletal proteins like myosin-IIA and negatively regulates the polymerization of its filaments. Under hypoxia conditions S100A4 activates the ERK signaling axis [14, 18]. S100A4 induces cell migration by disrupting molecular functions of the two molecules myosin-IIA and ERK.

Apart from β-catenin, S100A4 is transcriptionally regulated by SP1, AP1 and kappa recognition component (KRC) transcription factors and further it is controlled by CpG island methylation [19, 20]. S100A4 is overexpressed in a variety of cancer entities like CRC, non-small cell lung, breast, oesophageal, gastric and hepatocellular cancers. Moreover, its induced expression is positively correlated with tumor progression, metastasis and poor survival of patients [6, 21]. However, to our knowledge only miR-187-3p is reported to post-transcriptionally regulate S100A4 and S100A4 mediated metastasis [22]. This was so far only shown in hepatocellular carcinoma (HCC). However, there is no data about S100A4 post-transcriptional regulation by microRNAs (miRs), which might be down regulated due epigenetic silencing in CRC.

miRs are a class of small RNAs with lengths around 22 nucleotides (nt), which are expressed in a cell and tissue-specific manner. These molecules regulate mRNA stability and protein translation. miRs control a wide range of biological functions such as cellular proliferation, differentiation, apoptosis, EMT, migration, invasion, metastasis, drug resistance, epigenetic machinery and are also known as diagnostic and prognostic markers [23–25]. Especially, an upregulation of miR-21, and of the miR-29 family, as well as a downregulation of the miR-34 family and of miR-124a in tumor specimens are considered as prognostic markers in CRC [25–28]. DNA hypermethylation is one of the main reasons for the downregulation of the miR-34a family and miR-124a in cancer specimens [29, 30]. A change of the miR expression leads to a deregulation of their respective target proteins, which correlates with cancer progression.

All this evidence prompted us to design a comprehensive study on the post-transcriptional regulation of S100A4 by miRs and the epigenetic regulation of these miRs in CRC.

## RESULTS

### The S100A4-3′-UTR is a target of miR-505-5p and miR-520c-3p

**Figure 1:**
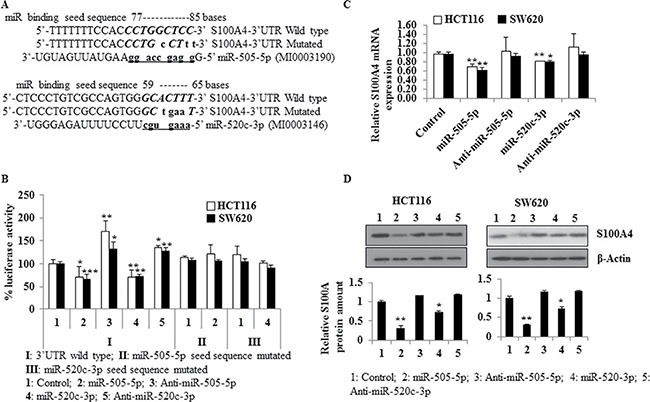
miR-505-5p and miR-520c-3p target the S100A4-3′UTR, and downregulate S100A4 expression (**A**) The seed sequences for miR-505-5p and miR-520c-3p in S100A4-3′-UTR (wild type) were detected by *in silico* predictions. The wild type and mutated seed sequence were cloned into the dual-luciferase vector and luciferase assays were performed to show direct regulation of the S100A4-3′-UTR by these miRs. (**B**) Luciferase reporter assays of HCT116 and SW620 cells transfected with control-miR (Control), miR-505-5p, anti-miR-505-5p, miR-520c-3p, anti-miR-520c-3p and co-transfected with either S100A4-3′-UTR wild type or the mutated constructs were performed. Renilla luciferase values of the vector were used for normalization. Percent luciferase activity was calculated based on the control values. (**C**) S100A4 mRNA expression was quantified using qRT-PCR and RPII was used as internal control. (**D**) S100A4 Western blotting was performed and bands were densitometrically quantified by normalizing to the internal control β-actin. Mean values of triplicates were represented. (*p < 0.05; **p < 0.01).

The 140 nt-3′UTR of S100A4 (#NM_019554.2) was screened for complementary seed sequences of miRs using *in silico* approaches (miRWalk, RNAhybrid). Complementary target sequences were identified for miR-505-5p at nt 77–85, and for miR-520c-3p at nt 59–65 (Figure 1A). The minimum free energy predicted for hybridization of miR-505-5p or miR-520c-3p with the S100A4-3′-UTR at their site is ΔG = –26.2 kcal/mol and ΔG = –24.5 kcal/mol, respectively, determined by mFold analysis (Supplementary Figure 1A, 1B).

Given the results from the *in silico* analyses, we hypothesized that the S100A4-3′-UTR could be a functional target of miR-505-5p and miR-520c-3p. To address this question, we cloned 164 nt of the 3′-UTR of S100A4, including 28 nt of the upstream coding sequence, in a pmirGLO dual-luciferase vector at the 3′-position of a luciferase reporter gene (S100A4-3′-UTR). The S100A4-3′-UTR was co-transfected along with control-miR, miR mimics or inhibitors (anti-miRs) of miR-505-5p and miR-520c-3p in HCT116 and SW620 cells. The luciferase activity of cells transfected with mimics together with S100A4-3′-UTR was significantly reduced compared to control-miR (HCT116 cells miR-505-5p (*p* = 0.02), miR-520c-3p (*p* = 0.01); SW620 cells miR-505-5p (*p* = 0.0009), miR-520c-3p (*p* = 0.003). On the other hand, cells that were transfected with the anti-miR inhibitor along with the S100A4-3′-UTR showed a significantly induced luciferase activity compared to the respective control (HCT116 cells anti-miR-505-5p (*p* = 0.01), anti-miR-520c-3p (*p* = 0.003); SW620 cells anti-miR-505-5p (*p* = 0.02), anti-miR-520c-3p (*p* = 0.005) Figure 1B). Similarly, co-transfections with constructs of S100A4-3′-UTR harboring the mutated seed sequences of either miR-505-5p or miR-520c-3p and the respective miRs did not significantly reduce the luciferase activity compared to the control-miR condition. Taken together, these data suggest that the 3′-UTR of S100A4 is a direct functional target of miR-505-5p and miR-520c-3p.

### miR-505-5p and miR-520c-3p regulate the S100A4 gene expression

To support the reporter assay results, HCT116 and SW620 cells were transfected with control-miR, miR mimics or anti-miRs of miR-505-5p and miR-520c-3p. The mRNA and protein expression analyses showed that miR-505-5p and miR-520c-3p reduced the S100A4-transcript levels in HCT116 (*p* = 0.01 and *p* = 0.01) and SW620 cells (*p* = 0.01 and *p* = 0.02), but no significant changes were observed in the anti-miR-505-5p and anti-miR-520c-3p conditions compared to the control-miR (Figure 1C). Western blotting confirmed the significant downregulation of S100A4 protein amounts in both cell lines, HCT116 and SW620, transfected with miR-505-5p (*p* = 0.01 and *p* = 0.04) and miR-520c-3p (*p* = 0.03 and *p* = 0.05). Moreover, transfection of anti-miR-505-5p and anti-miR-520c-3p slightly induced protein expression of S100A4 compared to the control-miR (Figure 1D, Supplementary Figure 5B). Taken together, these results suggest that S100A4 is post-transcriptionally regulated by miR-505-5p and miR-520c-3p through binding to its 3′-UTR in CRC cell lines.

### miR-505-5p and miR-520c-3p inhibit the S100A4-mediated migration and invasion

**Figure 2:**
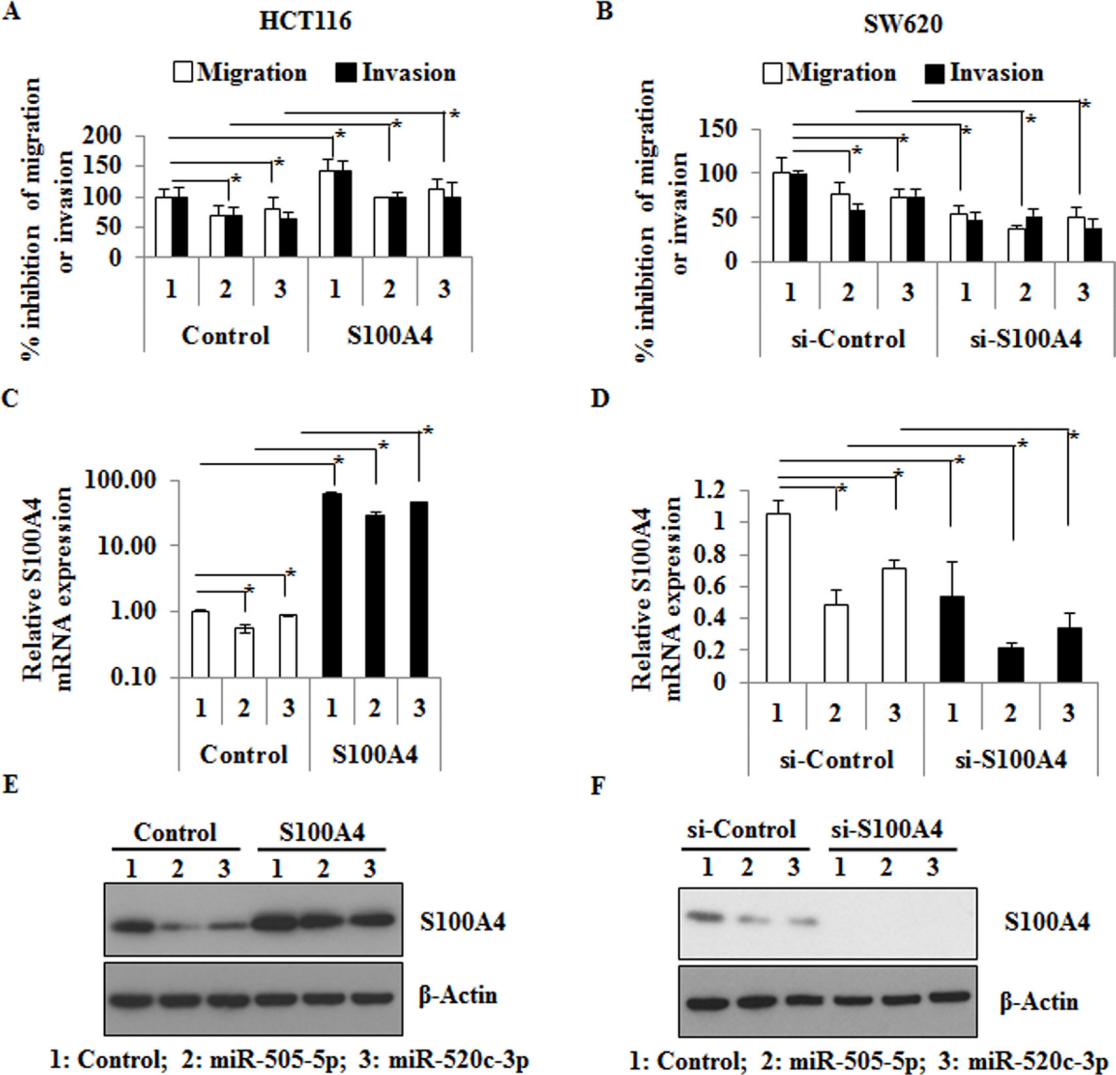
miR-505-5p and miR-520c-3p inhibit the S100A4 mediated migration and invasion *in vitro* (**A**) HCT116 cells were co-transfected with control-miR, miR-505-5p and miR-520c-3p along with either vector-control or -S100A4, respectively. After 48 h cells were plated on top of the Boyden chambers for migration and matrigel-coated Boyden chambers for invasion. After 16 h the migrated or invaded cells were measured as described in the materials and methods. (**B**) SW620 cells were co-transfected with control-miR, miR-505-5p and miR-520c-3p along with either si-RNA-control (si-control) or si-RNA-S100A4 (si-S100A4), respectively. After 48 h the cells were used for migration and invasion assay as stated above. (**C**, **D**) Transfection efficiency of ectopic overexpression and knock-down of S100A4 on mRNA level was analyzed using qRT-PCR. (**E**, **F**) S100A4 protein expression after transfection was analyzed by Western blots. RPII was used as internal control for S100A4 mRNA expression and β-actin for Western blotting. (*p < 0.05).

To further investigate the functional abilities of miR-505-5p and miR-520c-3p in mediating migration and invasion, we transfected HCT116 and SW620 cells with these two miRs separately. Migration (without matrigel) and invasion (with matrigel) assays were performed with Boyden chamber transwells. Ectopic overexpression of miR-505-5p and miR-520c-3p significantly reduced the migration (*p* = 0.01 and *p* = 0.05) and invasion (*p* = 0.01 and *p* = 0.02) in HCT116 as well as migration (*p* = 0.05 and *p* = 0.05) and invasion (*p* = 0.01 and *p* = 0.01) in SW620 cells (Figure 2A, 2B). In addition, we also showed the S100A4 specific effects by overexpression of S100A4 in combination with control-miR, miR-505-5p or miR-520c-3p, compared to the respective vector controls in HCT116 cells. Ectopic S100A4 expression significantly increased the migration and invasion in HCT116 cells compared to the vector control (*p* = 0.029 and *p* = 0.05; Figure 2A). S100A4 overexpression in combination with miR-505-5p or miR-520c-3p significantly increased migration (*p* = 0.006 and *p* = 0.02) and invasion (*p* = 0.006 and *p* = 0.03) of HCT116 cells compared to the miR overexpression in vector control cells, respectively. Additionally, we overexpressed miR-505-5p, miR-520c-3p or control-miR along with either si-RNA-S100A4 or si-RNA-control in SW620 cells. S100A4 knock-down significantly reduced the migration (*p* = 0.008) and invasion (*p <* 0.001) of SW620 cells compared to the respective control-siRNA (Figure 2B). Co-transfection of miR-505-5p or miR-520c-3p along with si-RNA-S100A4 significantly reduced migration (*p* = 0.001 and *p* = 0.04) and invasion (*p* = 0.05 and *p* = 0.007) compared to the miRs overexpression in the si-RNA-control cells (Figure 2B). Transfection efficiency of miR-505-5p or miR-520c-3p was determined by qRT-PCR (Supplementary Figure 1B, 1C). Ectopic overexpression or know-down of S100A4 and the impact of miRs on its mRNA and protein expression levels were analyzed by qRT-PCR and Western blot analysis, respectively (Figure 2C–2F). The endogenous expression of miR-505-5p and miR-520c-3p was lower in SW620 compared to HCT116 cells, whereas the S100A4 expression was higher (Supplementary Figure 3A; Supplementary Figure 2A, 2B; Supplementary Figure 4A). Therefore, HCT116 cells were transfected with anti-miR-505-5p, anti-miR-520c-3p and control-miR. Inhibition of the miRs led to increased S100A4 protein levels and induction of wound healing compared to the control-miR (Supplementary Figure 5).

Taken together, these results suggest that miR-505-5p and miR-520c-3p significantly inhibit S100A4-mediated migration and invasion in CRC cells.

### 5-Azacytidine treatment restores miR-520c-3p and inhibits the expression of S100A4

In order to explore further insights in the network of miR-505-5p and miR-520c-3p and their functional effects on S100A4 expression, we analyzed their epigenetic regulation. First, we determined their endogenous expression in a panel of CRC cell lines (Supplementary Figure 3A, Supplementary Figure 4A). The miRs were both expressed on a relatively low level (in comparison with other screened miRs, under similar conditions (data not shown)). miR-505 is located on chromosome Xq27.1 [–] as an intragenic miR of ATPase, Class VI, Type 11C (ATP11C) and may be expressed under the same promoter as its host gene. miR-520c is located on 19q13.42 [+] being part of a cluster of 46 miRs also known as chromosome 19 miRNA cluster (C19MC) [31]. The ATP11C and miR-505-5p expression were positively correlated in CRC cell lines (*R*^2^ =0.84, *p* = 0.05, Supplementary Figure 4B). Especially the miR-505-5p expression was higher in the noninvasive cell lines, Colo206f and Colo320DM, compared to the invasive SW620 and HCT116 cell lines (Supplementary Figure 4A) [30, 32]. Depending on their genetic location, we identified putative CpG islands in their promoter regions using *in silico* approaches. Furthermore, we determined their promoter methylation status using methylation-specific PCR (MSP) (Supplementary Figure 3B; Supplementary Figure 4C; Supplementary Figure 6A–6C). Additionally, we analyzed the host gene of miR-505, ATP11C in this study. The analyzed upstream regulatory region of miR-520c was rich in CpG islands having an average CG-content of more than 80%. Both, miR-505 and its host gene ATP11C, had no CpG-island in their upstream promoter region. However, several regions rich in individual CpG-sites were found that could be used for methylation studies (Supplementary Figure 6A–6C). Although most human promoters are associated with CpG-islands, this might not be the case for miR-505, which suggests a different regulation [33].

To analyze the degree of epigenetic regulation of the identified regions, gDNA was isolated from a panel of CRC cell lines and subjected to bisulfite conversion and subsequently MSP was performed with a specific set of “methylated” and “unmethylated” primers. The miR-505 upstream region was unmethylated, whereas the ATP11C upstream region was mostly methylated (Supplementary Figure 4C). Moreover, the miR-520c upstream regulatory region was completely hypermethylated in this cell line panel (Supplementary Figure 3B; Supplementary Figure 4C). Secondly, to functionally prove these MSP results, we have used the DNA-methyltransferase inhibitor 5-azacytidine (5-Aza). The treatment with 5-Aza had no significant impact on the expression level of miR-505-5p (Supplementary Figure 6D) in HCT116 and SW620 cell lines. Due to this limitation, we excluded miR-505-5p for further investigations.

**Figure 3:**
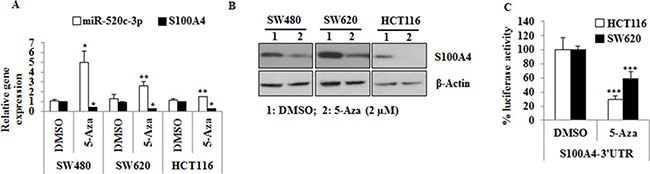
miR-520c regulatory region is hypermethylated in CRC cell lines and treatment with 5-Aza induces the expression of miR-520c-3p and downregulates S100A4 expression (**A**, **B**) SW480, SW620 and HCT116 cells were treated with 5-Aza (2 mM) for 3 days. qRT-PCR and Western blots were performed to analyze the impact of 5-Aza treatment on miR-520c-3p and S100A4 expression. RNUB6, RPII and β-actin served as internal controls. (**C**) HCT116 and SW620 cells transfected with the wild type S100A4-3′-UTR were treated with 5-Aza (2 μM) for 24 h and the luciferase activity was measured. Renilla luciferase activity was used for normalization. Percentage luciferase activity was significantly reduced after 5-Aza. (*p < 0.05).

On the other hand, the miR-520c-3p promoter was hypermethylated in all analyzed cell lines and its expression was significantly upregulated by treatment with 5-Aza in SW480 (*p* = 0.02), SW620 (*p* = 0.01) and HCT116 (*p* = 0.01) cells (Figure 3A). Moreover, the expression of its target gene S100A4 was downregulated at mRNA and protein level after 5-Aza treatment (Figure 3A, 3B). Secondly, the luciferase activity of S100A4-3′-UTR was significantly reduced upon 5-Aza treatment in SW620 (*p* = 0.001) and HCT116 (*p <* 0.001) cells (Figure 3C). Taken together, these results suggest that a restoration of miR-520c-3p could significantly inhibit the expression of S100A4.

### Combined bisulfite restriction analysis (COBRA) confirms hypermethylation of miR-520c in CRC cell lines

**Figure 4:**
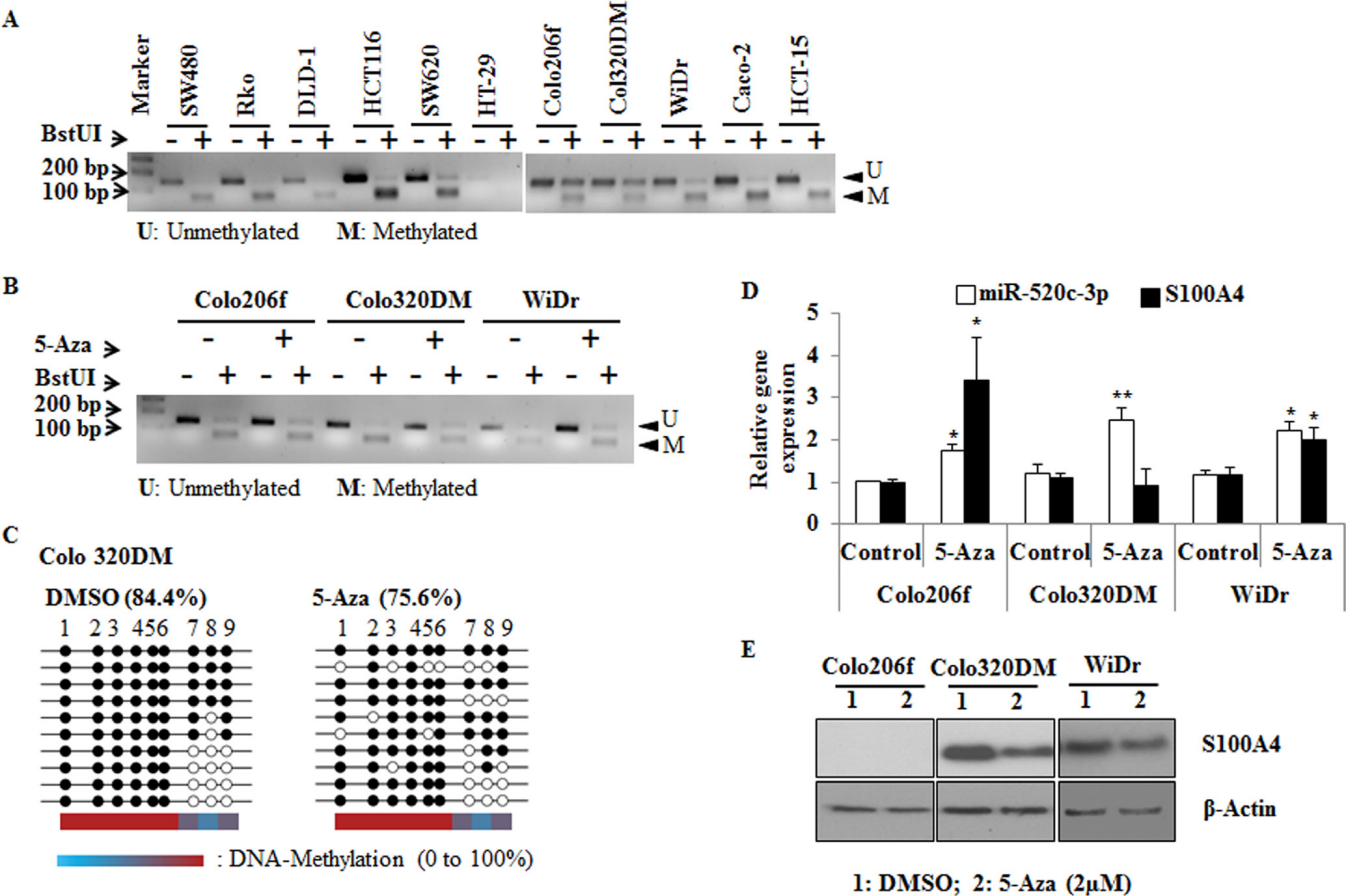
COBRA and bisulfite sequencing analysis of miR-520c regulatory region (**A**, **B**) COBRA analysis using the restriction enzyme BstUI was performed to estimate the methylation status of miR-520c-3p in CRC cell lines. Colo206f, Colo320DM and WiDr were also analyzed after 5-Aza (2 μm) treatment for 3 days. (**C**) Colo320DM cells were treated with 2 μM 5-Aza for 3 days and the methylation of miR-520c-3p was analyzed by bisulfite sequencing. From each group 10 representative clones were sequenced. Each circle represents CpG islands in the sequenced region, and the black circle represents the methylated and the empty white circle represents unmethylated CpG island. (**D**, **E**) qRT-PCR of miR-520c-3p, S100A4 and Western blot analysis of S100A4 were performed after 5-Aza treatment of Colo206f, Colo320DM for 3 days (20 μg of total protein used for Western blot) and WiDr (5 μg of total protein used for Western blot) cell lines (from low to moderate S100A4 expressing cells). RNUB6, RPII and β-actin served as internal controls. (*p < 0.05; **p < 0.01).

To further illustrate the hypermethylation of the regulatory region of miR-520c, COBRA analysis was performed. For this assay, bisulfite converted gDNA of CRC cell lines was amplified by non-methylation specific PCR and then digested with the restriction enzyme BstUI. The enzyme recognizes specifically CpG-sites that were not modified by the bisulfite treatment and therefore were methylated before. The PCR of the miR-520c regulatory element created a 152 bp amplicon. BstUI cuts the PCR product in a 67 bp and an 85 bp fragment, which was visible as a new band on an agarose gel. In most of the analyzed cell lines the regulatory region of miR-520c was methylated (Figure 4A). However, the less invasive cell lines Colo206f, Colo320DM and WiDr were partly unmethylated [30]. Comparatively, these cell lines had less or moderate S100A4 protein amounts compared to SW620 cells (Supplementary Figure 2B). To have additional evidence about the methylation status of the regulatory region of miR-520c and S100A4 especially in these three cell lines, the cells were treated with 5-Aza for 3 days and subsequently DNA, RNA and proteins were isolated and investigated. COBRA analysis and bisulfite sequencing showed a reduced CpG-island methylation of the miR-520c regulatory element after 5-Aza treatment in these three cell lines. The reduction of methylated CpGs was almost 10% in Colo320DM cells compared to the DMSO treated control cells (Figure 4B, 4C).

Nakamura et al, reported that hypermethylation of the S100A4 gene promoter correlates with its activation, expression and also with the cell invading capacity [32]. In this report the authors showed that after 5-Aza treatment S100A4 mRNA levels were increased in the less invasive cell lines SW837, LoVo and DLD-1 but not in WiDr. However, in our study we used Colo206f, Colo320DM and WiDr cell lines, which also showed a relatively low invasive phenotype. Treatment with 5-Aza induced the expression of S100A4 (except in Colo320DM) and its post-transcriptional regulator miR-520c-3p, which was determined by qRT-PCR (Figure 4D). Further, a clear reduction of S100A4 protein expression was observed in Colo320DM and WiDr cells compared to the DMSO condition, but no S100A4 protein was detectable in Colo206f cells (Figure 4E). This confirmatory analysis suggests that epigenetic silencing of miR-520c-3p is an important mechanism during cancer progression and could be one of the reasons for S100A4 upregulation in CRC cell lines.

### The miR-520c regulatory region is hypermethylated in CRC tumor specimens

**Figure 5:**
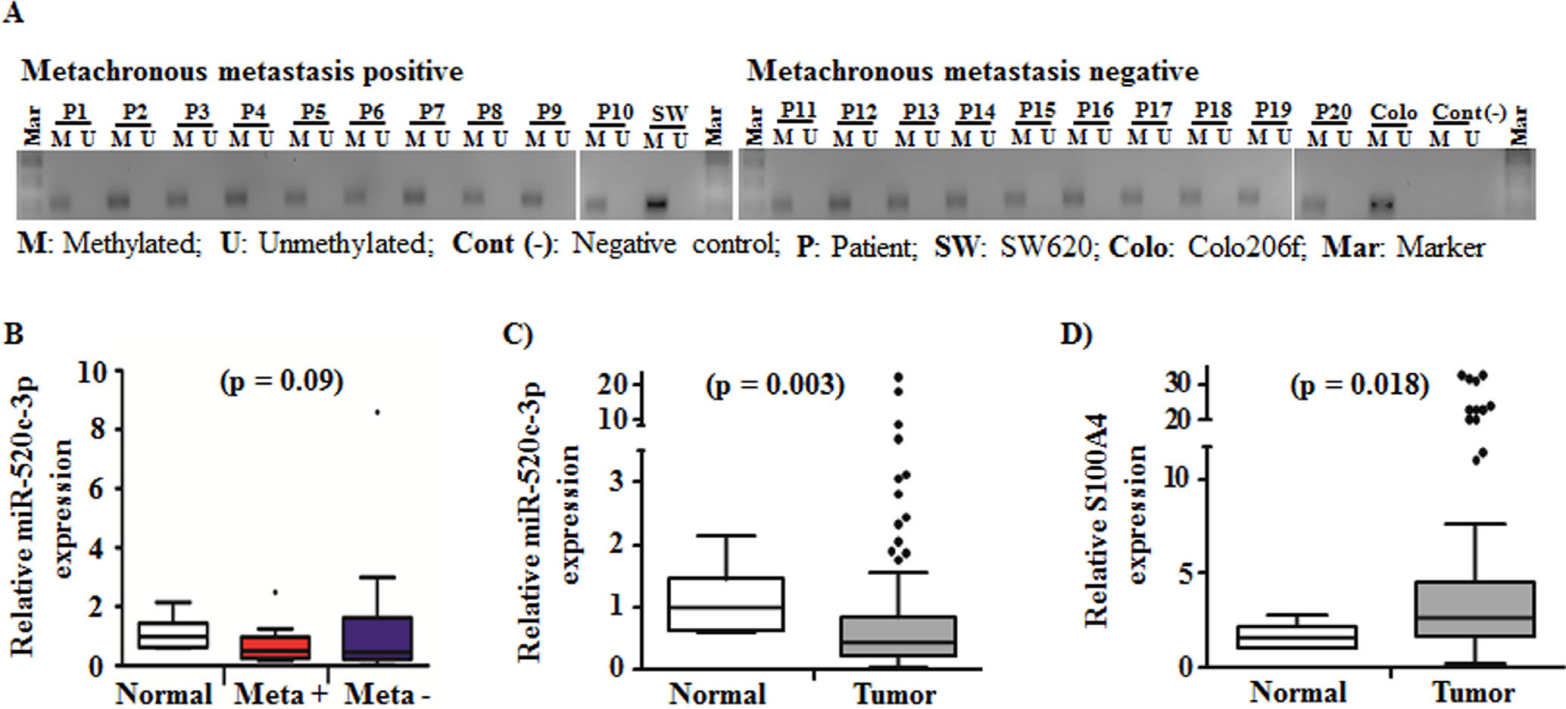
Methylation status of the miR-520c regulatory region and expression of miR-520c-3p and its target S100A4 in a cohort of CRC tumor specimens (**A**) Methylation specific PCR products of metachronous metastasis positive and metachronous negative CRC tumor specimens were analyzed using agarose gel electrophoresis. The miR-520c regulatory element was methylated in all specimens. The two cell lines SW620 and Colo206f served as internal controls. (**B**) miR-520c-3p expression in metachronous metastasis positive (*n* = 10) and negative (*n* = 10) CRC tumor specimens in comparison to representative normal mucosa were quantified using qRT-PCR. (**C**, **D**) miR-520c-3p and S100A4 expression in CRC tumor specimens in comparison to representative normal mucosa were quantified using qRT-PCR. RPII and RNUB6 served as internal controls.

In order to further determine the epigenetic regulation mechanisms of miR-520c in CRC patient tumors, we used representative tumor specimens of our cohort, which were either positive or negative for metachronous metastasis. Comparable to the CRC cell lines, the miR-520c regulatory region was hypermethylated irrespective to the metachronous metastasis status (Figure 5A). The miR-520c-3p expression showed a trend for downregulation in specimens derived from metachronous metastasis positive tumors compared to metachronous metastasis negative tumor specimens (Figure 5B). Moreover, in a cohort of 59 CRC patient tumor specimens miR-520c-3p was significantly downregulated (*p* = 0.003) compared to the normal mucosa tissues and its target S100A4 was significantly upregulated (*p* = 0.018, Figure 5C, 5D). Taken together, these methylation analysis and gene expression studies revealed that due to epigenetic downregulation of miR-520c-3p its post-transcriptional target S100A4 was upregulated in CRC tumor specimens.

### miR-520c-3p inhibits metastasis formation in mice

**Figure 6:**
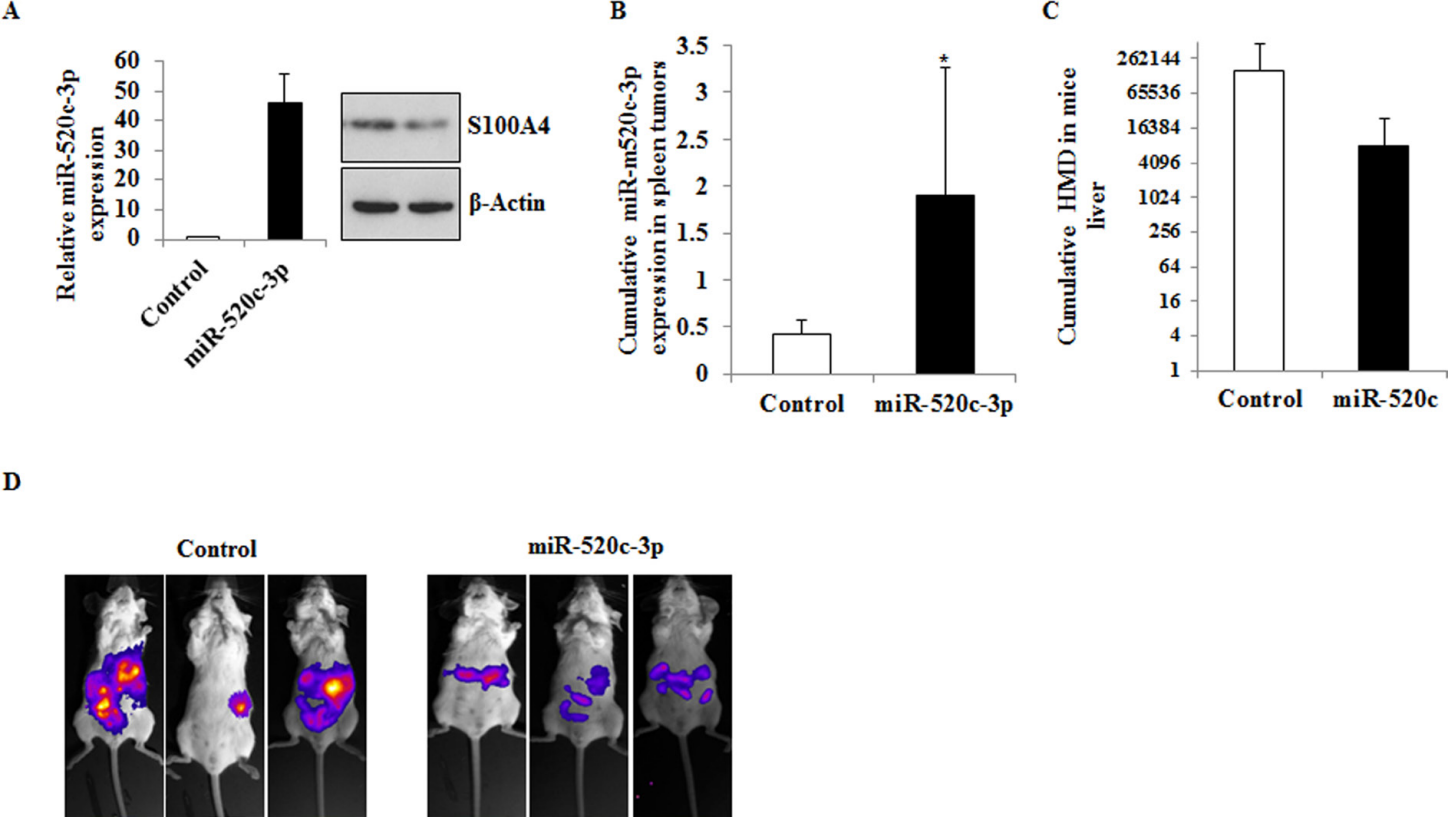
Overexpression of miR-520c-3p inhibits metastasis formation *in vivo* (**A**) HCT116 cells stably expressing miR-520c-3p or control-miR were generated. The overexpression of the miR was quantified by qRT-PCR and compared to control-miR. The expression of the target gene S100A4 in the miR overexpressing cells was downregulated as indicated by Western blotting. (**B**) The cells were intrasplenically injected in SCID beige mice and sacrificed after 20 days. The expression of miR-520c-3p in the shock-frozen primary tumor in the spleen was quantified by qRT-PCR. (**C**) Metastasized cells into the liver of the animals were analyzed using specific primers to exclusively detect HMD by qRT-PCR. Less amounts of HMD were detectable in the liver sections of the miR-520c-3p overexpressing group. (**D**) The cells were intrasplenically injected in SCID beige mice and tumor and metastasis formation was monitored by bioluminescence imaging. Representative mice from each group are shown on the day the animals were sacrificed. (*p < 0.05).

To further investigate the *in vivo* relevance of miR-520c-3p, HCT116 cells stably transduced with either miR-520c-3p (HCT116-LUC-miR-520c-3p) or control-miR (HCT116-LUC-Control) were intrasplenically injected in SCID beige mice. Before intrasplenic injection, the stable expression of miR-520c-3p and downregulation of its target S100A4 were analyzed by qRT-PCR and Western blot (Figure 6A). The mice were monitored by bioluminescence once per week and a similar procedure was performed before the termination of the experiment. Mice injected with HCT116 cells stably overexpressing miR-520c-3p (HCT116-LUC-miR-520c-3p) (*p* = 0.009; Figure 6B) showed slightly reduced amounts of human microsatellite DNA (HMD) in their liver tissue compared to the control group (HCT116-LUC-Control) (Figure 6C) and also we observed less metastatic lesions in the miR-520c-3p expressing group (Figure 6D). However, these differences were not statistically significant due to the less number of sample size.

Collectively, these data show that migration, invasion, and metastasis formation *in vivo* are inhibited by miR-520c-3p overexpression.

## DISCUSSION

Here we investigated the post-transcriptional regulation of the important metastasis-associated gene S100A4 by miRs and their epigenetic regulation in CRC. In this study, we identified two miRs, miR-505-5p and miR-520c-3p, which post-transcriptionally inhibit the expression of S100A4, thereby hindering its mediated migration, invasion and formation of distant metastasis of CRC cells [34]. Moreover, especially the expression of miR-520c-3p was downregulated in CRC cell lines and tumor specimens, which is partially caused by epigenetic silencing of the regulatory region of miR-520c-3p. Furthermore, our study extends the existing knowledge about epigenetic regulation of S100A4 by promoter hypermethylation in CRC cell lines [32].

In this study, by *in silico* analyses we have identified miR-505-5p and miR-520c-3p as potential candidates targeting the 3′UTR of S100A4. By co-transfecting miR mimics together with the S100A4-3′-UTR harboring either the wild type or mutated seed sequences of the predicted miRs, we identified S100A4 as a novel target of miR-505-5p and miR-520c-3p. Moreover, ectopic expression of miR-505-5p or miR-520c-3p decreased S100A4 mRNA and protein expression, whereas inhibition of the endogenous miRs by anti-miRs increased S100A4 expression. Further, loss or gain of function of miR-505-5p and miR-520c-3p along with S100A4 overexpression or knock-down clearly demonstrated that they mitigate the S100A4 mediated migration and invasion [35–37]. In the majority of CRC tumor specimens Wnt signalling is known to be dysregulated and therefore constitutively active, whereas S100A4 is a downstream effector molecule of this signalling [9, 34]. Secondly, loss of epigenetic control leads to increased expression of S100A4, which was shown to be a prognostic marker for poor survival of CRC patients [5, 38]. Beside the reports about its transcriptional regulation the post-transcriptional regulation of S100A4 is not well understood. miR-187-3p is reported to post-transcriptionally regulate S100A4 and S100A4-mediated metastasis in hepatocellular carcinoma (HCC) [22]. Here we demonstrate that loss of post-transcriptional regulation of S100A4 due to epigenetic silencing of miR-505-5p and miR-520c-3p expression could be one of the main causes for the induction of S100A4 expression in CRC and other cancer entities.

Epigenetic regulation of gene activity plays a major role in cancer development and disease progression [39]. Canonical DNA methylation occurs mainly within the context of CpG-dinucleotides that are organized in bigger regions termed islands or shores [40]. It is already an established mechanism during the embryonal period in a tissue specific manner [41]. The DNA methyltransferase 1 (Dnmt1), Dnmt3a and Dnmt3b enzymes are required for the establishment and maintenance of these methylation patterns [42]. Loss or gain of function of methyltransferases result in abnormal gene regulation, which could lead to downregulation of tumor suppressor genes and upregulation of tumor promoting genes [30, 39]. Nakamura et al. reported for the first time that S100A4 promoter hypermethylation leads to its downregulation in less aggressive colon adenocarcinoma cell lines [32]. In concordance with these findings, we also observed induced S100A4 mRNA expression after 5-Aza treatment in less aggressive CRC cell lines such as Colo206f, Colo320DM and WiDr, but not in the more aggressive cell lines SW480, SW620 and HCT116. However, S100A4 protein amounts were reduced after 5-Aza treatment in all of these cell lines irrespective of their invasive or aggressive nature, except for Colo206f cells [30]. In comparison to the other screened CRC cell lines, Colo206f and Colo320DM had less or no endogenous expression of S100A4. In particular, Colo206f showed induced S100A4 mRNA expression after 5-Aza treatment. However, in Western blotting even at higher concentrations of protein loading we didn't observe any S100A4 protein band compared to the Colo320DM cell line. One explanation could be that S100A4 might be regulated in a cell line specific manner via a different mechanism other than discussed.

5-Aza treatment increased the miR-520c-3p expression in a panel of CRC cell lines irrespective of their characteristics. This gives additional evidence to analyze the hypermethylation status of the miR-520c regulatory region in all CRC cell lines. COBRA and bisulfite sequencing analysis further confirmed that the miR-520c regulatory region is hypermethylated in CRC cell lines. This finding is in line with previously described epigenetic silencing of the C19MC cluster members in HCC and gastric cancer [43, 44]. Loss of methylation in its regulatory region increases the miR-520c-3p expression and inhibits its target gene S100A4 protein amount after 5-Aza-treament in comparison to DMSO controls. On the other hand, the methylation pattern of the miR-520c regulatory region did not show significant differences in CRC tumor specimens, which were either positive or negative for metachronous metastasis. Interestingly, we have observed that the miR-520c-3p expression tend to be lower expressed in metachronous metastasis positive specimens compared to the negative specimens. These results suggest that besides CpG hypermethylation additional alternative epigenetic or transcriptional regulatory mechanisms might be involved in expression control of miR-520c-3p. Overall, in a cohort of CRC patient tumor specimens, a significant downregulation of miR-520c-3p expression and upregulation of S100A4 mRNA expression was observed. About miR-520c-3p there are several reports indicating its role as tumor suppressor miR or oncomir depending on the cancer entity [45–48]. Our findings in this study strongly support miR-520c-3p as tumor suppressor miRNA which is downregulated in CRC and also causes upregulation of its target proteins such as the metastasis-associated gene S100A4 in tumor tissue [9, 37, 49]. Moreover, miR-520c-3p belongs to the C19MC cluster and also other members of this cluster were reported to have a tumor suppressive function, which is in line with previous findings that miR family members can regulate similar targets and pathways [49–53]. To sum up, our findings highlight the key role of miR-520c-3p as a tumor suppressor, which is silenced by hypermethylation of its regulatory region in CRC. Loss of miR-520c-3p expression during tumorigenesis is in part responsible for the S100A4 upregulation in CRC. Our study also identified S100A4 as the first cancer-associated target of miR-505-5p and its functional impact on metastasis-associated characteristics like migration and invasion. miR-505-5p was so far only shown to be dysregulated in cancer tissue compared to normal mucosa but no functional evidence was reported [54, 55].

S100A4 is known for inducing proliferation, EMT, migration and invasion *in vitro* and tumor metastasis events *in vivo* [56, 57]. sh-RNA mediated knock-down of S100A4 significantly reduced the CRC cell mediated metastasis formation [57]. With this line of evidence, we intrasplenically injected HCT116 cells stably overexpressing miR-520c-3p in SCID beige mice and observed slightly reduced metastasis formation in the liver compared to the control-miR group. With this results, one can speculate that especially in this context the downregulation of miR-520c-3p due to a hypermethylation of its regulatory region could be one of the mechanisms for S100A4 overexpression in different cancer entities, apart from its previously described epigenetic and Wnt/β-catenin mediated transcriptional regulation [14, 58].

In conclusion, this study highlights a pivotal role of miR-505-5p and miR-520c-3p in various aspects of tumorigenesis such as migration, invasion, and *in vivo* metastasis formation in CRC through the post-transcriptional regulation of their novel target S100A4. Additionally, we demonstrated the epigenetic silencing of miR-520c-3p as possible mechanism during carcinogenesis, which lead to upregulation of S100A4. These findings certainly add additional information to the S100A4 biology and its targeted therapeutic approaches.

## MATERIALS AND METHODS

### Construction of 3′-UTR-luciferase plasmids, reporter assays, miRs, siRNA and plasmids

The 3′-UTR of S100A4 (including 28 nt of the coding sequence and 136 nt of 3-′UTR) was amplified using cDNA from SW480 cells and cloned into the XhoI and SalI sites of the pmirGLO dual-luciferase miRNA target expression vector (#E1330, Promega, USA), checked for orientation, sequenced and named S100A4-3′-UTR. Specific miR-505-5p and miR-520c-3p seed sequences were mutated using PCR based site directed mutagenesis kit (#210518, Agilent technologies, USA). Cloning and site directed mutagenesis primers are provided in Supplementary Table 1 and 2. For reporter assays, cells were co-transfected using Lipofectamine 2000 (Invitrogen, Carlsbad, USA) with 1 μg of luciferase construct along with 50 nM of control-miR or mimics/inhibitor miRs. Reporter assays were performed 48 h post-transfection using the dual-luciferase assay-system, normalized for transfection efficiency by renilla luciferase.

miRNA mimic miR-505-5p (ID: MC12497), miR-520c-3p (ID: MC12719), miRNA inhibitor anti-miR-505-5p (ID: MH12497), anti-miR-520c-3p (ID: MH12719), miR-negative control (#4464058), siRNA targeting S100A4 (ID:12226) and scrambled control (#4390843) were purchased from Ambion, USA. Lentiviral constructs of control-miR (#CmiR0001-MR03) and miR-520c-3p (#HmiR0327-MR03) were purchased from GeneCopoeia, USA. CMV promoter–controlled S100A4 cDNA and the empty vector as control were kind gifts from Claus Heizmann (University of Zurich, Switzerland).

### Cell lines, cultures and drugs

Human CRC cell lines (SW480, Rko, DLD-1, HCT116, SW620, HT-29, Colo320DM, WiDr, Caco-2 and HCT-15) were purchased from American Type Culture Collection (ATCC; Manassas, VA), and Colo206f from German Collection of Microorganisms and Cell culture (DSMZ Leibniz Institute; Braunschweig, Germany). The cells were grown in a humidified incubator at 37°C and 5% CO_2_ with RPMI (Colo320DM, Colo206f, SW480, WiDr, HCT-15) and DMEM (rest of the cells used for this study) media supplemented with 10% fetal calf serum (FCS). All used cell lines were authenticated using genotyping and were mycoplasma free. Original stock solutions of 5-Aza-2′-deoxycytidine (5-Aza-dC (#A2385), Sigma Chemical Co., USA) at a concentration of 10 mM was stored at –20°C and freshly dissolved in culture medium before use.

### Virus production and generation of stably miR expressing cell lines

1.5 × 10^7^ HEK293 cells were plated for transfection. After 24 h cells were transfected using 2.85 ml of serum free medium, which was mixed with 90 μg of polyethylenimine (PEI) and kept at room temperature for 5 min. Either 30 μg of control-miR or miR-520c-3p lentiviral plasmids (containing also GFP) with packing vectors (20 μg psPax2, 10 μg pMD2.G) were individually mixed and incubated at room temperature for 20 min. These mixtures were added to the respective plates. After 48 h of incubation, the supernatant was collected and filtered (0.45 μm filter). In a further step the filtered supernatant was loaded on a 20% sucrose cushion and centrifuged at 4°C for 4 h in an ultracentrifuge (28,000 rpm). The viral particles were dissolved in 500 μl sterile PBS and stored at -80°C. HCT116 cells were transduced in 6 well plates with a multiplicity of infection (MOI) less than 10 for each respective well. After 24 h of incubation, the virus containing medium was replaced with the regular medium and the GFP expressing cells were sorted using FACS. After sorting, the miR-520-3p expression of the stably overexpressing HCT116 cells were analyzed by qRT-PCR and compared to the control-miR cells. In addition, control-miR and miR-520c-3p expressing cells were transduced with eGFP-LUC virus. The miR-scrambled (HCT116-LUC-Control) and miR-520c-3p (HCT116-LUC-miR-520c-3p) along with luciferase-GFP stably expressing cells were used for animal experiments. The detectable luminescence values of both cell lines were comparable.

### Metastasis formation in xenografted mice

Animal experiments were performed at EPO GmbH, Berlin, in accordance with the UKCCCR guidelines and approved by the responsible local authorities (State Office of Health and Social Affairs, Berlin, Germany). For analysis, 3 × 10^6^ cells of HCT116-LUC-Control or HCT116-LUC-miR-520c were transplanted into the spleens of 8 weeks old severe combined immunodeficiency (SCID) beige mice (randomly assigned to 6 mice per group before transplantation). The mice were imaged once a week in a NightOWL LB 981 system (Berthold Technologies, Germany) by intraperitoneal injection of 150 mg/kg D-luciferin (Biosynth, Switzerland) under temporal anesthesia with isoflurane (Sigma-Aldrich, Germany). Mice were sacrificed 20 days after tumor cell transplantation. Spleens (site of tumor injection) and livers (metastasis target organ) were removed and shock-frozen in liquid nitrogen and cryosections were performed for isolation of genomic DNA (Qiagen, Hilden, Germany) and RNA (TRIzol, Invitrogen). The level of liver metastasis was evaluated by quantifying HMD using PCR with primers provided in Supplementary Table 3. ImageJ was used for color coding of luminescence signal intensity of human cells.

### Patients and samples

Fresh snap-frozen surgical specimens of tumor tissues and representative corresponding normal specimens from 59 CRC patients were collected with informed written consent (approved by Charité Ethics Committee, Charité-Universitätsmedizin, Berlin), preserved and processed as explained in our previous publications [59]. The main patients’ characteristics are reported in Supplementary Table 4. None of the patients received preoperative chemo/radiation therapy and none of the researchers conducting gene expression and statistical analyses had access to disclosed clinical-pathological data.

### DNA/RNA/Protein isolation and cDNA synthesis from cells and fresh snap-frozen CRC specimens

DNA, RNA and protein isolation and cDNA synthesis were performed as described previously by Ilm et al. [59]. Briefly, for isolation of DNA and RNA from frozen tissues, cryosections were performed and every fifth section was stained with hematoxylin. Tumor cell areas were evaluated and marked by a pathologist. Tumor cells were removed from the unstained slides and further proceeded according to the manufactures protocol for isolation of DNA and RNA. Expression of mature miR-505-5p (MS00009849), miR-520c-3p (MS00007413) and U6-snRNA (RNUB6) (MS00033740) were determined by the miScript SYBR Green PCR Kit (Qiagen), and normalized using the 2^–ΔΔCt^ method relative to RNUB6. S100A4 and RPII expression was also quantified as described above and RPII served as normalization control. Primers are provided in Supplementary Table 5. Western blot analysis was performed using specific antibodies for S100A4 (#Dako-A5114) or β-actin (#SIGMA-A1978).

### Cell migration and invasion assay

These assays were performed as described previously by Ilm et al. [59]. Briefly, cells were seeded in 6-well plates and transfected with 50 nM control, miR-505-5p, miR-520c-3p or anti-miR-505-5p, miR-520c-3p (Thermo Fisher Scientific) using Lipofectamine RNAiMAX Reagent (Thermo Fisher Scientific) according to the manufacturer's instructions. After 48 h of incubation the cells were seeded for migration and invasion assays. 16 h later the migrated or invaded cells were quantified using Luminescent Cell Viability Assay (CellTiter-Glo®, Promega). These assays were performed three independent times, each in technical triplicates.

### 5-Aza-2′-Deoxycytidine (5-aza) treatment of cells, bisulfite conversion of DNA and methylation and sequencing analysis

5′-Aza treatment, bisulfite conversion and methylation analyses (PCR was performed using HotStarTaq Plus DNA Polymerase (#203605) from Qiagen, Germany) were performed as described previously [59]. CpG islands upstream of the TSS (Transcription start site) or pri-miR start site were determined with the CpG island searcher (http://www.uscnorris.com/cpgislands2/cpg.aspx), and PCR primers were designed using the Methprimer software (http://www.urogene.org/methprimer). The primer sequences are provided in Supplementary Table 6.

A bisulfite-sequencing PCR was done for the 152 bp long GC-rich region upstream of miR-520c. Converted DNA was amplified by PCR and the products were used for TOPO TA cloning kit (Invitrogen). Ten clones were sequenced from each cell line using T3 primer (sequence: AATTAACCCTCACTAAAGGG, LGC Biotech, Germany).

### Combined bisulfite restriction analysis (COBRA)

COBRA was used to analyze the methylation status of the regulatory element of miR-520c. The primers were picked using MethPrimer via the CpG-island prediction function. Primer sequences are provided in Supplementary Table 7. The AmpliTaq Gold polymerase was used for the PCR. The PCR product was cleaned up using QIAquick PCR Purification kit following the standard protocol. The restriction enzyme BstUI was used for the analysis based on the recognition site. It cuts the sequence CGCG within the 152 bp PCR product at position 85, consequently creating one 67 bp and one 85 bp fragment. The digestion was performed in a thermocycler for 1 h at 60°C. A second control reaction was performed without enzyme for each sample. The whole sample was then loaded on a 3% agarose gel.

### Statistical analysis

Statistical analysis was performed using GraphPad Prism 5. The non-parametric Mann-Whitney-U test was applied to calculate *p*-values for patients to compare two groups. The non-parametric Kruskal-Wallis test was applied for more than two groups. For all other data analysis, the comparison of two different groups was done by Student's *t-test*. The Pearson model was used for correlation analysis. For all tests a *p-value* < 0.05 was considered as statistically significant. The whiskers of a box plot show 1.5 times the interquartile range according to the definition of Tukey.

## SUPPLEMENTARY MATERIALS FIGURES AND TABLES


