# Supplementary material for: Epigenetic silencing of miR-520c leads to induced S100A4 expression and its mediated colorectal cancer progression
Source: Oncotarget. 2017 Feb 18;8(13):21081–94. doi: 10.18632/oncotarget.15499 (PMC5400567; doi:10.18632/oncotarget.15499)
Supplement: Supplementary file 1 [file oncotarget-08-21081-s001.pdf]

Epigenetic silencing of miR-520c leads to induced S100A4 expression and its mediated colorectal cancer progression

Supplementary Materials

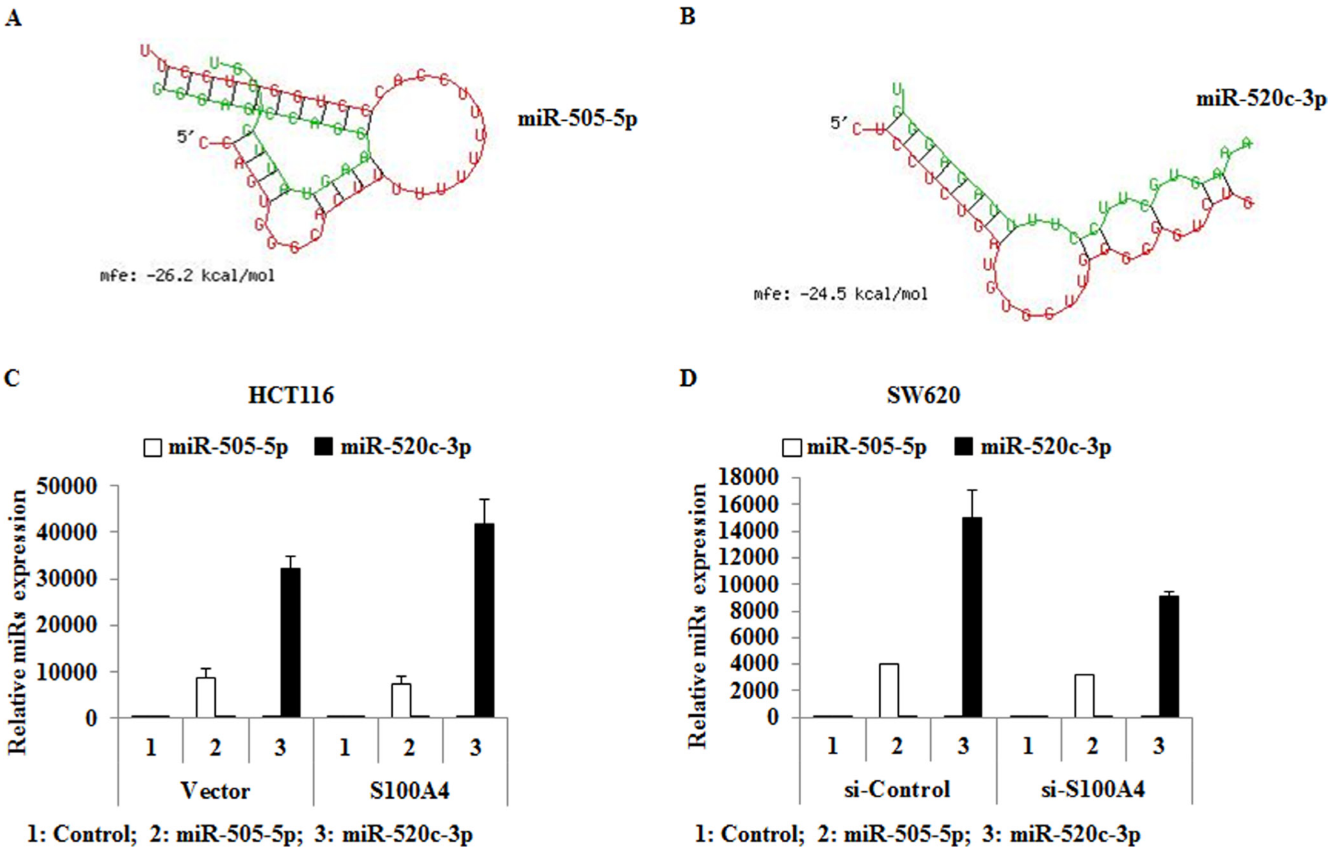

Supplementary Figure 1: Secondary structure prediction (RNAhybrid) of the binding between the seed sequences of miR-505-5p and miR-520c-3p within the S100A4-3′-UTR and ectopic overexpression of miR-505-5p and miR-520c-3p. (**A**, **B**) The S100A4-3′-UTR contains seed sequences for miR-505-5p (green-highlighted sequence) at position 77 to 85 nt and miR-520c-3p (green-highlighted sequence) position 59 to 65 nt and showed a strong hybridization energy ΔG of -26.2 kcal/mol and -24.5 kcal/mol, respectively. (**C**, **D**) HCT116 or SW620 cells were transfected with control-miR, miR-505-5p, miR-520c-3p along with either vector-control or -S100A4 expression (HCT116) or si-Control and si–S100A4 (SW620). Transfection efficiency of miRs was measured using qRT-PCR, whereas RNUB6 served as internal control.

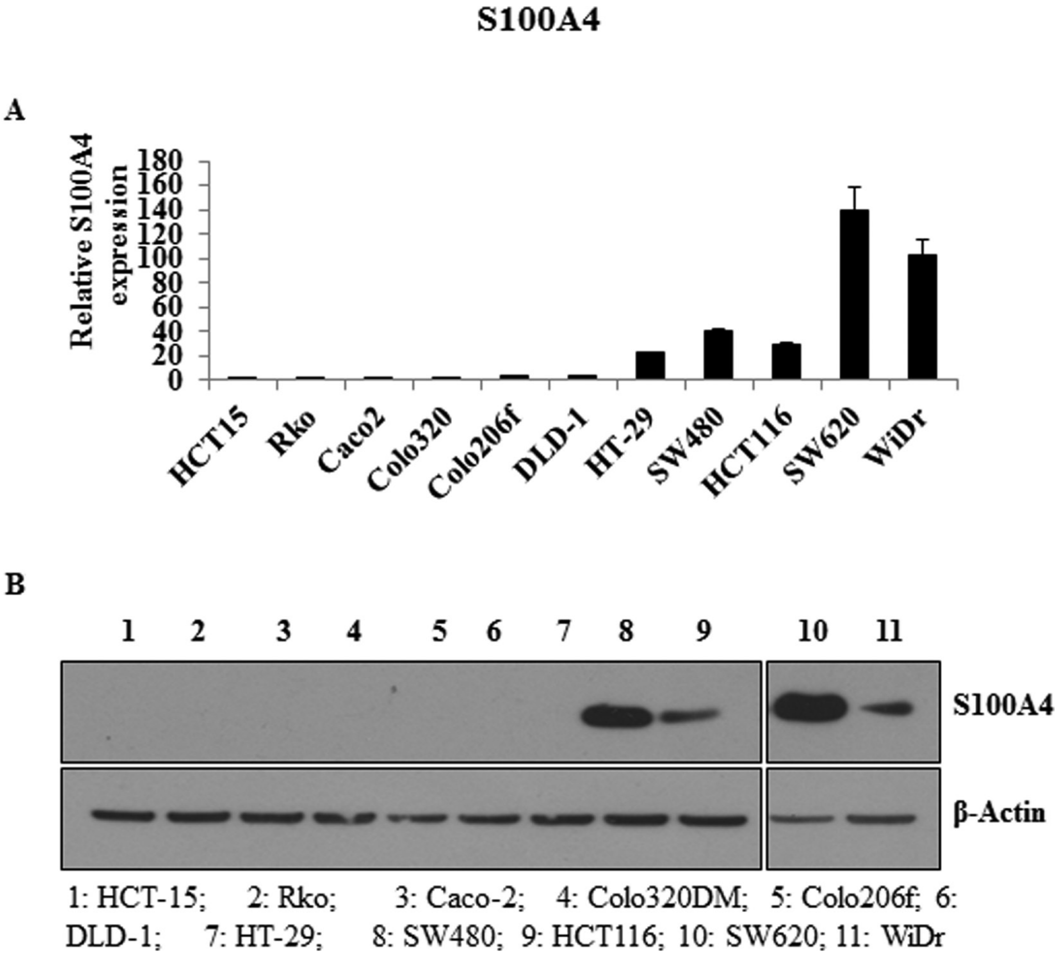

Supplementary Figure 2: S100A4 endogenous mRNA expression and protein amount in CRC cell lines. (**A**) S100A4 expression in a panel of CRC cell lines was analyzed by qRT-PCR, whereas RPII served as internal control. (**B**) S100A4 protein amounts were screened in the same panel of CRC cell lines, whereas β-actin served as internal control (Samples were analyzed using two gels, whereas sample preparation, gel electrophoresis, blotting and development were simultaneously performed).

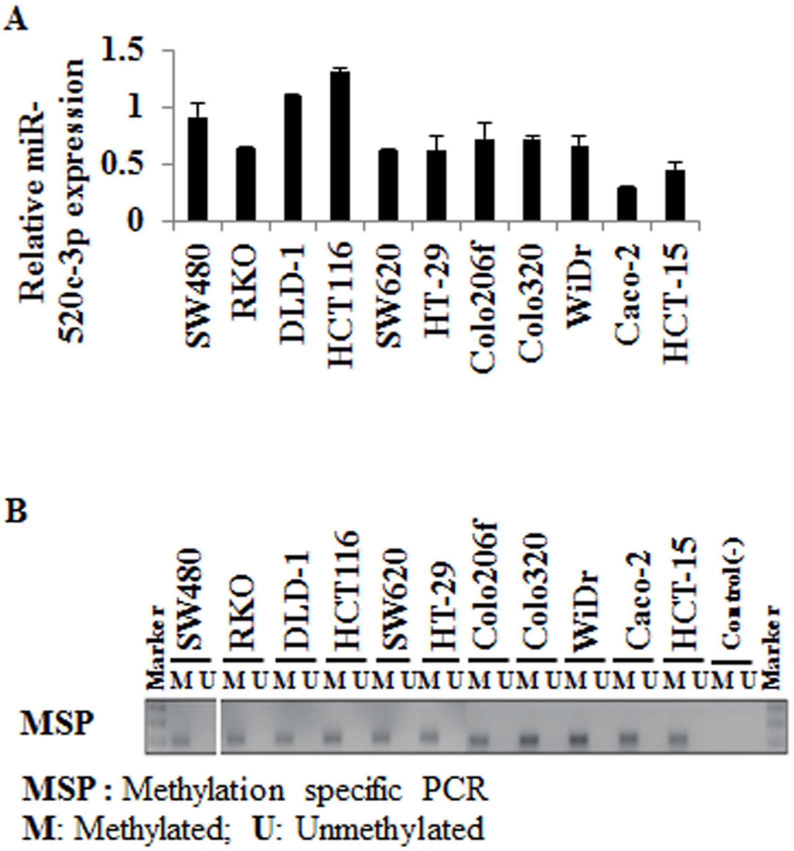

Supplementary Figure 3: miR-520c expression and methylation status in a panel of CRC cell lines. (**A**) Endogenous miR-520c-3p expression was quantified using qRT-PCR. RNUB6 served as internal control. (**B**) Results of the methylation-specific PCR of the CRC cell lines were analyzed using gel electrophoresis of the PCR products.

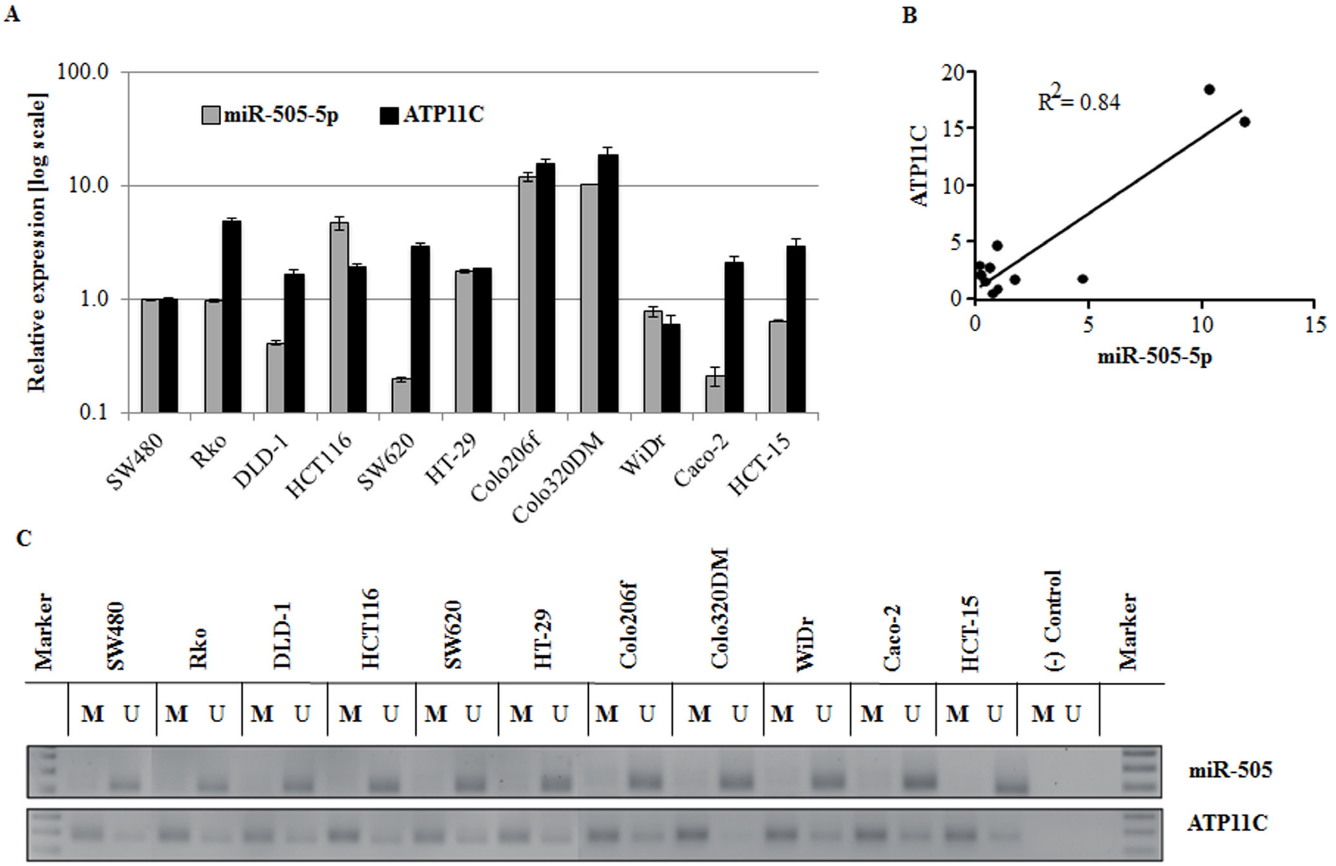

Supplementary Figure 4:miR-505-5p and its host gene ATP11C expression, and methylation status in a panel of CRC cell lines. (**A**, **B**) Relative miR-505-5p and ATP11C expression levels in a panel of CRC cell lines were analyzed by qRT-PCR, whereas RNUB6 and RPII served as internal controls. miR-505-5p and ATP11C expression levels positively correlated in CRC cell lines ($R^2 = 0.84$). (**C**) PCR products of CRC cell lines obtained from methylation-specific PCR for the possible regulatory region of miR-505 and its host gene ATP11C were analyzed by agarose gel electrophoresis.

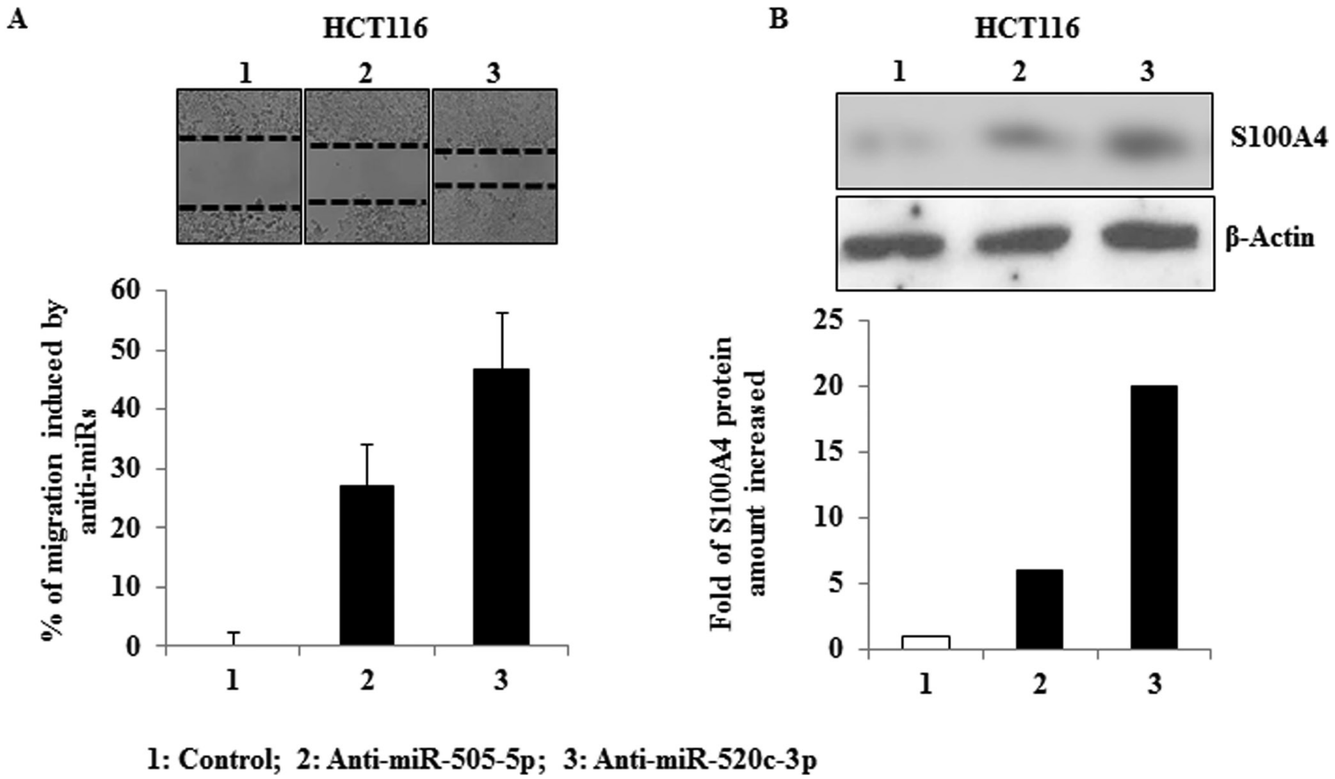

Supplementary Figure 5: Inhibition of miR-520c-3p significantly induces wound healing and S100A4 protein expression in HCT116 cells. (**A**) HCT116 cells were transfected with anti-miR-505-5p and anti-miR-520-3p. After 48 h cells were plated in wound healing culture plates (#80241, Ibidi, Germany) and incubated for 24 h to allow the cells to attach. The cell separating plastic covers were removed. After 24 h of incubation the wound was photographed under a brightfield microscope. Wound healing was measured comparative to control and the bar graph shows the relative migration (wound healing) induced by anti-miR-505-5p and anti-miR-520c-3p. (**B**) After 48 h, protein was isolated from anti-miR transfected cells and S100A4 protein expression was analyzed using Western blot (presented here is the shortest exposure to show the clear differences), whereas β-actin served as internal control. The bar graph shows the densitometric analysis of S100A4 protein in comparison to the control.

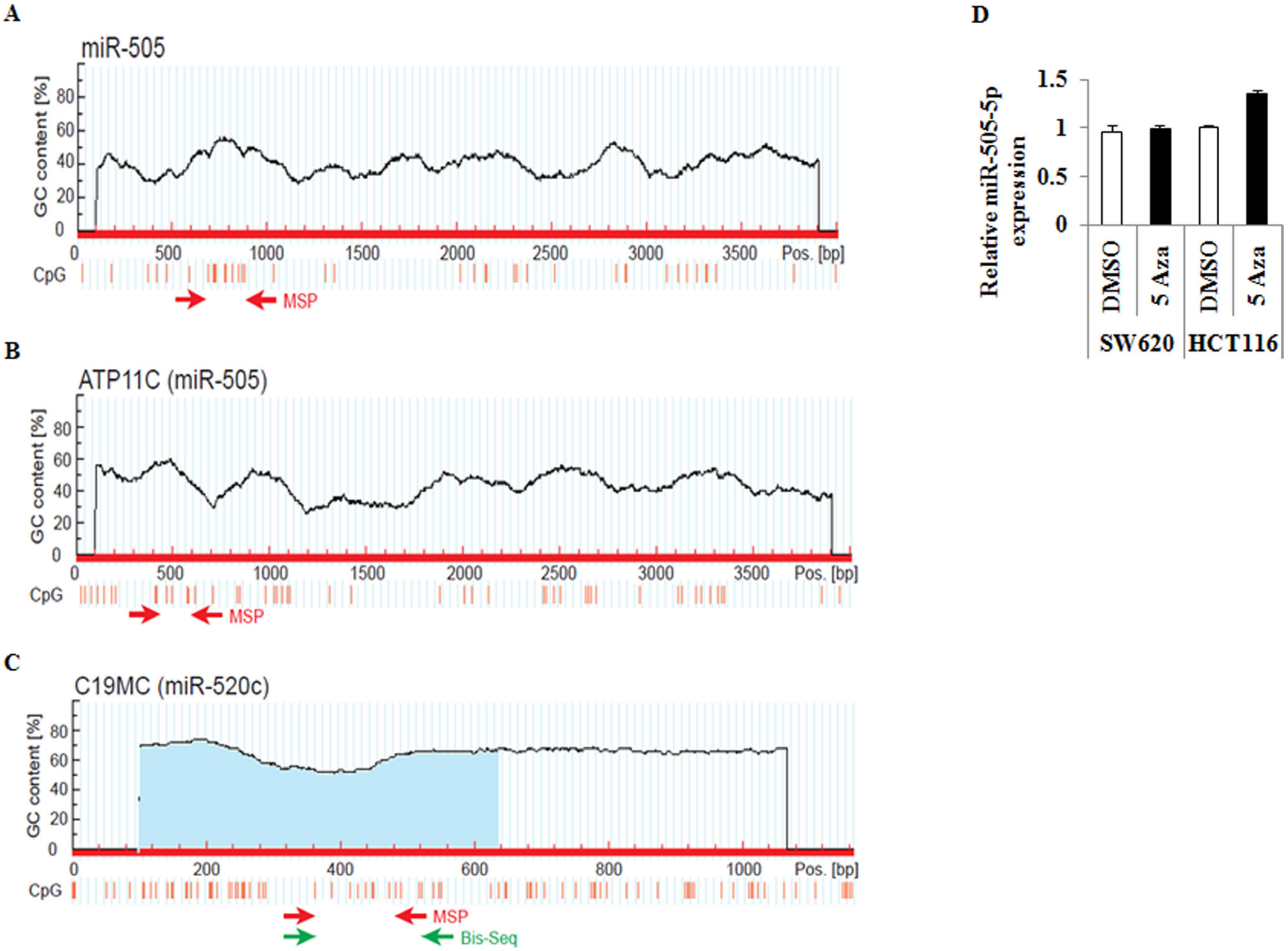

Supplementary Figure 6: Diagrammatic representation of CpG-islands in the upstream regions of miR-520c, miR-505 and the promoter region of its host gene ATP11C. (**A–C**) The Methprimer tool was used to predict CpG islands in upstream regions of miR-505, ATP11C and miR-520c. For miR-520c a reported region was analyzed, whereas for miR-505 and ATP11C the 4 kb upstream region was used for methylation analysis. Primer locations for methylation specific PCR and bisulfite sequencing are represented by arrows in red (MSP) and green (Bis-Seq). (**D**) The expression of miR-505-5p and ATP11C after 5-Aza (2 μM) treatment for 3 days was analyzed in SW620 and HCT116 cells using qRT-PCR.

Supplementary Table 1: Oligos used for S100A4-3′-UTR cloning

No	Oligo Name	Sequence (5′ --- 3′)
1	S100A4_UTR_For	5′ CCCTCGAGCTTCCCAGATAAGCAGCCCA 3′
2	S100A4_UTR_Rev	5′ CCGTCGACCTTCCAAGAATCTTTATTGAAC 3′

Supplementary Table 2: Oligos used to mutate miR-505 and miR-520c seed sequences at S100A4-3′-UTR

No	Oligo Name	Sequence (5′ --- 3′)
1	S100A4_m505_For	AGCCAGGGTGGAAAAAAAATTCAGCCCACTGGCGACAGGGAG
2	S100A4_m505_Rev	CTCCCTGTCGCCAGTGGGCTGAATTTTTTTTCCACCCTGGCT
3	S100A4_m520c_For	CAGCATCAAGCACGTGTCTGAAAAGGCCAGGGTG GAAAAAAAAAAGT
4	S100A4_m520c_Rev	ACTTTTTTTTTTCCACCCTGGCCTTTTCAGACACGTGCTTGATGCTG

Supplementary Table 3: Oligos used for measuring the Human microsatellite DNA (HMD) in mice liver tissue

No	Gene Name	Sequence (5′ --- 3′)
1	HMD_F	GGGATAATTTCAGCTGACTAAACAG
2	HMD_R	AAACGTCCACTTGCAGATTCTAG

Supplementary Table 4: Patients information

		Total	Percentage
**Sex**	M	36	61%
	F	23	39%
**Age median (min to max)**	63.0 (37.9 to 87.8)		
**UICC (7th Edition)**	I	17	29%
	II	22	37%
	III	20	34%
**pT**	1	6	10%
	2	16	27%
	3	27	46%
	4	10	17%
**pN**	0	39	66%
	1	14	24%
	2	6	10%
**Metachronous metastases**	no	35	59%
	yes	24	41%

Clinico-pathological features of the 59 CRC patients.
pT = Pathologic tumor classification. pN = Pathologic lymph node status.

Supplementary Table 5: Oligos used for S100A4 and RPII screening by qRT-PCR

No	Gene Name	Sequence (5′ --- 3′)
1	S100A4_For	TGTGATGGTGTCCACCTTCC
2	S100A4_Rev	CCTGTTGCTGTCCAAGTTGC
3	RPII _For	GCACCACGTCCAATGACAT
4	RPII _Rev	GTGCGGCTGCTTCCATAA

Supplementary Table 6: Oligos used for methylation-specific PCR (MSP) of miR-505 and miR-520c

No	Gene Name	Sequence (5′ --- 3′)
1	miR-505_M_F	ATTTAGGTTGGAGTGTAATGGTACG
2	miR-505_M_R	TTAAAAAACCGAAATAAAAAAATCG
3	miR-505_U_F	TTTAGGTTGGAGTGTAATGGTATGA
4	miR-505_U_R	AAAAAACCAAAATAAAAAAATCACC
5	miR-520c_M_F	CGGTAAAATTTTGAATTTTTTTGTC
6	miR-520c_M_R	AAAATACATACTAACCTACCGCGTT
7	miR-520c_U_R	TGGTAAAATTTTGAATTTTTTTGTTG
8	miR-520c_U_R	AAAATACATACTAACCTACCACATT

M: Methylated; U: Unmethylated.

Supplementary Table 7: Oligos used for Bisulfite sequencing and COBRA analysis of miR-520c

No	Gene Name	Sequence (5′ --- 3′)
1	miR-505_Bis_F	GGTAAAATTTTGAATTTTTTTGT
2	miR-505_Bis_R	AAAAAAACCTATTAACTAAAAACCC
